# Enhancing Early Detection of Alzheimer’s Disease: An Ensemble Model for Multi-Domain Cognitive Assessment Using Voice and Video

**DOI:** 10.3390/s26123833

**Published:** 2026-06-16

**Authors:** Shinwoo Ham, Donghun Min, Hyo Jin Jon, Jung Eun Shin, Eun Yi Kim

**Affiliations:** 1 Voinosis Inc., Seoul 05029, Republic of Korea; swham@voinosis.com (S.H.); dhmin@voinosis.com (D.M.); hjjon@voinosis.com (H.J.J.); eardoc70@voinosis.com (J.E.S.); 2 Artificial Intelligence & Computer Vision Laboratory, Konkuk University, Seoul 05029, Republic of Korea

**Keywords:** Alzheimer’s disease, early detection, multi-modal, multi-domain, ensemble, deep learning

## Abstract

Accurate early screening of Alzheimer’s disease (AD) is crucial, yet traditional diagnostic methods are often limited by invasiveness or high costs. Therefore, there is a critical need for non-invasive biomarkers that enable precise and accessible screening. In this study, we propose a multi-modal digital biomarker framework designed to accurately detect AD by evaluating impairments across multiple cognitive domains, such as language, working memory, and visuospatial attention. By leveraging voice and video data, our approach significantly enhances user accessibility and real-world applicability. We validated the proposed framework using a dataset of 128 participants, comprising 77 healthy controls (HCs) and 51 patients with AD. While individual cognitive tasks yielded F1-scores ranging from 69.23% to 77.78% and sensitivities from 69.23% to 80.77%, our ensemble strategy significantly enhanced detection performance, achieving an F1-score of 83.64% and a sensitivity of 88.46%. These findings confirm that the proposed multi-modal digital biomarker framework, enhanced via ensembling, provides a highly accurate, scalable, and practical solution for the non-invasive screening and detection of AD.

## 1. Introduction

Dementia refers to an acquired syndrome in which cognitive abilities—including memory, language, reasoning, and the capacity to carry out everyday tasks—decline progressively and to a degree that meaningfully disrupts a person’s independence and quality of life [1]. Among its many causes, Alzheimer’s disease (AD) remains the most prevalent, accounting for roughly 60 to 80 percent of all diagnosed cases [2]. Given AD’s irreversible nature, early detection is critical for enabling timely intervention and slowing disease progression [3]. However, conventional diagnostic approaches, including magnetic resonance imaging (MRI) and positron emission tomography (PET), often require specialized equipment and expert clinical interpretation, limiting their accessibility and scalability in real-world settings [4,5]. These challenges have driven growing interest in practical, non-invasive behavioral biomarkers that can be collected using everyday devices.

Among the various accessible behavioral signals, speech has emerged as a particularly attractive modality due to its strong association with cognitive processes. Speech production inherently involves language formulation, memory retrieval, and executive control, all of which are affected by AD [6,7]. Early research primarily explored linguistic features derived from spontaneous speech, revealing that AD patients exhibit reduced lexical diversity, simplified syntax, and increased semantic errors [8,9]. However, linguistic features are inherently language-dependent, limiting their generalizability across diverse populations. To address this limitation, subsequent studies have investigated acoustic patterns, such as speech rate, pause duration, prosody, and pitch variability, which provide language-agnostic indicators of cognitive decline and can be extracted without transcription [10].

In parallel, eye movement has gained attention as a complementary behavioral biomarker. Cognitive impairment in AD affects visual attention and oculomotor control, leading to measurable abnormalities such as prolonged fixations, reduced saccade amplitudes, and inefficient visual exploration patterns [11,12,13]. Eye-tracking approaches offer several advantages, including minimal dependence on language and robustness to environmental noise. Furthermore, recent deep learning-based gaze estimation methods enable accurate tracking using standard webcams, significantly improving accessibility and practicality.

Despite these advances, most existing approaches rely on a single task, capturing only a partial view of the complex and heterogeneous nature of AD. In practice, cognitive impairment manifests differently across individuals, affecting multiple domains such as memory, language, and executive function to varying degrees [14]. As a result, single-task approaches are inherently limited and may fail to detect certain patient groups, increasing the risk of missed diagnoses. From a screening perspective, minimizing false negatives (i.e., avoiding missed AD cases) is particularly critical.

To address this limitation, we propose a multi-domain behavioral sensing framework for AD detection that integrates complementary behavioral signals across multiple cognitive domains. Specifically, participants perform three tasks: Counting Backward (executive function), Picture Description (verbal communication), and Gaze Tracking (oculomotor control). For each task, dedicated deep learning models extract task-specific behavioral biomarkers and independently assess AD likelihood. These task-wise predictions are then integrated into a unified diagnostic decision, enabling robust characterization of impairments across multiple cognitive domains. By leveraging complementary sensing pathways, the proposed framework explicitly targets the reduction in missed AD cases, offering a more sensitive and reliable paradigm for real-world, non-invasive screening.

Our proposed framework is validated on a cohort of 128 participants, including 77 healthy controls (HC) and 51 AD patients. Individual sensing tasks exhibit heterogeneous diagnostic performance, with F1-scores ranging from 69.23% to 77.78% and sensitivities from 69.23% to 80.77%, indicating that no single task sufficiently captures the full spectrum of cognitive impairment. In contrast, by integrating complementary sensing signals across tasks, the proposed framework substantially enhances diagnostic reliability. The ensemble achieves an F1-score of 83.64% and, notably, a sensitivity of 88.46%, demonstrating a significant reduction in missed AD cases. Furthermore, a conservative ensemble strategy prioritizing sensitivity attains 92.31%, reinforcing the framework’s effectiveness in minimizing false negatives. These findings underscore that multi-domain integration is essential for robust detection, establishing our framework as a practical, non-invasive, and scalable solution with high potential for real-world Alzheimer’s disease screening and accessible evaluation.

The remainder of this paper is organized as follows. Section 2 reviews related studies, and Section 3 describes the study participants and clinical assessments. Section 4 details the proposed methodology. Section 5 presents the experimental results and discussion. Finally, Section 6 concludes the paper.

## 2. Related Work

### 2.1. Alzheimer’s Disease

Alzheimer’s disease, the most prevalent cause of dementia, is a progressive neurodegenerative disorder that evolves from preclinical stages to mild cognitive impairment (MCI) and ultimately dementia. AD leads to severe impairments in memory, language, and executive function, substantially diminishing patients’ quality of life. In 2018, an estimated 50 million people worldwide were living with dementia, and this number is projected to rise to 152 million by 2050 [15]. The associated economic burden already exceeds one trillion US dollars annually and is expected to double within the next decade [16].

Because AD accounts for the majority of dementia cases, its detection still largely depends on conventional dementia screening workflows. Standard clinical assessment typically combines structured cognitive testing, functional ability evaluation, neurological examination, laboratory workup to exclude reversible causes, and brain imaging. Among commonly used screening tools, the Montreal Cognitive Assessment (MoCA) performs reasonably well for MCI detection, with reported sensitivities ranging from 80% to 100% at the conventional 25/26 cutpoint [17]. In contrast, the Mini-Mental State Examination (MMSE), although still widely adopted, remains substantially less sensitive for subtle cognitive impairment [17]. More comprehensive neuropsychological batteries can improve diagnostic accuracy. However, these traditional methods suffer from several important limitations, including dependence on trained clinicians, lengthy administration time, substantial cost, and restricted accessibility outside specialist settings. These limitations have accelerated the development of scalable biomarker-based approaches for AD diagnosis.

### 2.2. Unimodal Sensing for AD Detection

Neuroimaging has been a cornerstone of AD detection, with MRI and PET serving as the most widely used modalities. MRI provides detailed structural information, particularly in distinguishing between gray and white matter, while PET captures functional abnormalities such as glucose hypometabolism and pathological protein accumulation. Early automated approaches relied on classical machine learning methods, including LDA [18], logistic regression [19], and SVM [20], applied to hand-crafted features, but were limited in modeling the high-dimensional complexity of neuroimaging data. The emergence of deep learning significantly improved performance, with 2D CNNs learning discriminative features from individual slices [21], followed by 3D CNNs that better capture volumetric spatial relationships [22]. Despite these advances, neuroimaging-based approaches rely on specialized equipment and clinical infrastructure, which limits their accessibility for large-scale and longitudinal screening applications. These limitations have motivated the exploration of alternative biomarkers, including electrophysiological, blood-based, and behavioral modalities.

Beyond neuroimaging, electrophysiological and biosignal-based approaches have also been explored for AD detection. Electroencephalography (EEG), in particular, has attracted considerable attention due to its ability to capture neural dynamics associated with cognitive decline. Previous studies have reported that EEG-based biomarkers can reflect abnormalities in brain connectivity, spectral power, and neural synchronization related to AD progression [23,24,25]. Recent deep learning approaches further improved automated analysis by leveraging temporal and spatial representations from multichannel EEG recordings [26,27]. However, EEG-based systems typically require dedicated acquisition hardware, controlled recording environments, and extensive preprocessing to mitigate motion artifacts and noise, which may limit their practicality in large-scale or home-based screening scenarios.

More recently, blood-based biomarkers have emerged as a promising direction for early AD detection. Biomarkers such as amyloid-beta (Aβ), phosphorylated tau (p-tau), and neurofilament light chain (NfL) have demonstrated strong associations with AD pathology [28,29,30]. Advances in assay sensitivity and AI-based analysis have further accelerated the development of blood-based diagnostic frameworks [31,32]. Nevertheless, despite their clinical potential, blood biomarkers still require invasive sampling procedures and laboratory-dependent analyses, which may reduce their suitability for continuous or frequent monitoring applications.

Speech has emerged as a highly accessible biomarker, as cognitive decline is reflected in both language production and speech dynamics [6,7,33]. Prior studies have explored both open-ended and fixed speech tasks to capture complementary aspects of cognitive impairment. Open-ended tasks, such as picture description, are effective for revealing lexical-semantic deficits, including reduced vocabulary richness, vagueness, and increased hesitations [8,9]. In contrast, fixed cognitive tasks, such as counting backward or reading, impose controlled cognitive load, making temporal and prosodic abnormalities-such as delayed initiation, increased pauses, and instability in speech rate-more explicitly observable. However, linguistic feature-based approaches remain language-dependent and rely on accurate transcription, limiting their generalizability and scalability [34,35]. To address these limitations, recent work has shifted toward acoustic patterns directly extracted from raw speech signals, including prosody, pause duration, speech rate, and spectral characteristics [10,36]. These acoustic features enable fully automated, language-agnostic analysis while effectively capturing cognitive impairment, making speech-based AD detection more practical for real-world, non-invasive screening.

Additionally, behavioral sensing (e.g., gaze tracking) provides critical insights into visuospatial attention and executive control, as cognitive decline is reflected in abnormalities in visual attention and oculomotor control. Early eye-tracking studies analyzed fixation duration, saccadic amplitude, scanpath efficiency, and dwell-time patterns during structured tasks such as reading, visual search, and scene viewing [37,38,39,40]. These studies consistently reported that patients with AD exhibit prolonged fixation durations, reduced exploratory behavior, and impaired attentional shifts compared with healthy controls, indicating deficits in visual information processing. As a sensing modality, gaze offers key advantages, including minimal dependence on language and robustness to acoustic or environmental noise. Unlike traditional eye-tracking methods that rely on dedicated infrared cameras, recent vision-based gaze estimation techniques enable accurate gaze tracking using standard cameras alone, allowing scalable and non-invasive assessment [41,42]. These characteristics make gaze a complementary sensing signal for capturing cognitive impairment, particularly in domains not directly observable through speech.

However, relying on a single modality or a single assessment task inevitably encounters limitations. A single-task evaluation often fails to encompass the multifaceted heterogeneity of cognitive decline. Patients with higher cognitive reserves may successfully compensate for deficits in a specific domain, allowing them to perform relatively normally [43,44]. As a result, deficits may remain undetected under a single-task condition, increasing the risk of false-negative diagnoses and potentially delaying timely clinical intervention.

### 2.3. Multi-Modal and Multi-Task Sensing for AD Detection

To overcome the inherent limitations of single-task sensing, recent research has increasingly explored multi-modal and multi-task frameworks for AD detection. A large body of work has focused on multi-modal fusion within the neuroimaging domain, most notably combining MRI and PET to jointly capture structural and metabolic abnormalities [45,46,47]. While such approaches achieve strong diagnostic performance, they primarily reflect static snapshots of brain pathology and are unable to capture dynamic, real-time cognitive impairments that emerge during task execution [48,49]. Moreover, their reliance on specialized equipment limits their applicability for scalable and continuous screening [14,50].

To address these challenges, more recent studies have investigated behavioral multi-sensing approaches, integrating modalities such as speech and gaze to capture cognitive dynamics in a non-invasive manner. These approaches consistently demonstrate improved performance over unimodal methods [51,52,53], suggesting that complementary sensing signals provide synergistic diagnostic value. However, a key limitation is that many of these frameworks operate within a single cognitive task (e.g., combining speech and gaze during picture description), which restricts their ability to capture the heterogeneous nature of AD. In practice, patients may exhibit selective preservation in certain cognitive domains, allowing deficits to remain undetected when assessed under a single task condition [14,54,55].

To effectively minimize missed AD cases, it is therefore essential to jointly assess multiple cognitive domains. By integrating complementary behavioral signals elicited from distinct task paradigms, a more comprehensive and reliable characterization of cognitive impairment can be achieved. While recent advances have explored cross-modal attention and hierarchical fusion strategies, they remain largely constrained by task homogeneity. In contrast, our approach explicitly adopts a heterogeneous multi-task design in which each task probes a distinct cognitive domain, enabling the integration of complementary behavioral evidence across diverse cognitive functions. By simultaneously assessing these domains, the proposed framework enhances sensitivity, enabling more robust detection of Alzheimer’s disease in real-world screening scenarios.

## 3. Materials

This section describes the study participants and the clinical assessment procedures. The study protocol was approved by the Institutional Review Board of Konkuk University Medical Center (No. 2024-02-040). All procedures were conducted in accordance with the ethical principles of the Declaration of Helsinki (2013 revision) and the International Council for Harmonisation Good Clinical Practice (ICH-GCP) guidelines.

All participants received a detailed explanation of the study objectives and procedures from the research staff and provided written informed consent prior to participation. Participants were informed that the study involved the collection of both voice and facial video data during task performance. Due to privacy considerations, participants were given the option to decline video recording while still participating in the speech-based tasks.

### 3.1. Participants

This study included a total of 128 participants recruited at Konkuk University Hospital from February 2025 to December 2025. Some participants declined video recording due to privacy concerns; therefore, they completed only the speech-based tasks and did not perform the gaze-based task. Table 1 summarizes the demographic characteristics of the study participants, including age, education level, gender, and Mini-Mental State Examination (MMSE) scores. The MMSE [56] is a widely used screening instrument for dementia, with scores ranging from 0 to 30, where higher scores indicate better cognitive function.

### 3.2. Data Acquisition

Participants performed the tasks in a clinical setting at the hospital under the supervision of medical personnel using a 24-inch monitor (OT2410W, Advance One D&T Co., Ltd., Seoul, Republic of Korea). Speech signals were recorded using a HyperX Cloud II headset (HyperX, Fountain Valley, CA, USA) with a sampling rate of 44,100 Hz. While traditional eye-tracking systems require specialized hardware, recent advances in deep learning have made it possible to estimate gaze from facial videos. Accordingly, video data were captured using a Logitech C922x Pro Stream Webcam (Logitech International S.A., Lausanne, Switzerland) at 30 fps and stored at a resolution of 640 × 480 to improve storage efficiency.

### 3.3. Clinical Assessment

The dataset used in this study was collected under expert clinical supervision. Alzheimer’s disease (AD) diagnoses were established by board-certified psychiatrists through comprehensive clinical assessments, including the Mini-Mental State Examination (MMSE) [56], neuropsychological testing, activities of daily living evaluations (the Korean Instrumental activities of daily living) [57], behavioral assessments using the Caregiver-Administered Neuropsychiatric Inventory [58], and dementia severity ratings based on the Clinical Dementia Rating (CDR) [59]. To facilitate early-stage AD screening, participants with mild cognitive impairment (MCI) are included in the AD group. Participants with depressive symptoms, impaired mobility, neurological disorders, or major psychiatric illnesses according to the Diagnostic and Statistical Manual of Mental Disorders, Fourth Edition (DSM-IV) [60], were excluded.

### 3.4. Task Design

**Figure 1:**
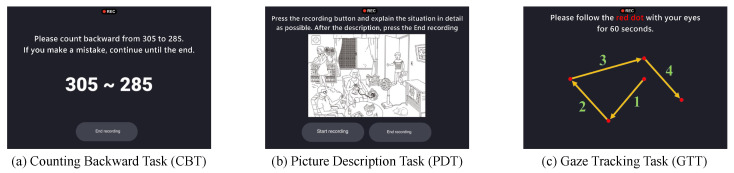
Description of the clinical tasks. Participants were asked to perform each task following the examples shown above. In the Gaze Tracking Task (GTT), the red dots indicate target positions, the yellow arrows represent the movement trajectory of the target, and the numbers denote the sequential order of target presentation.

To assess participants’ cognitive abilities from multiple perspectives, we designed three tasks: two speech-based tasks, the Counting Backward Task (CBT) and the Picture Description Task (PDT), and a gaze-based task, the Gaze Tracking Task (GTT). An overview of the three tasks is illustrated in Figure 1. For each task, task-specific features are extracted.

- Counting Backward Task (CBT): Participants count backward from 305 to 285. This task is designed to measure attention and working memory and has been commonly used in assessing cognitive decline, particularly in elderly patients with dementia.
- Picture Description Task (PDT): Participants describe a pre-selected photograph in their own words. This task is primarily used to assess aphasia and other higher cortical functions, providing insight into expressive abilities.
- Gaze Tracking Task (GTT): Participants follow a red dot as it moves across a screen. This task is designed to assess visual attention and oculomotor control by analyzing gaze behavior and head movements, which can reflect impairments in attentional regulation and visuomotor coordination.

## 4. Methods

**Figure 2:**
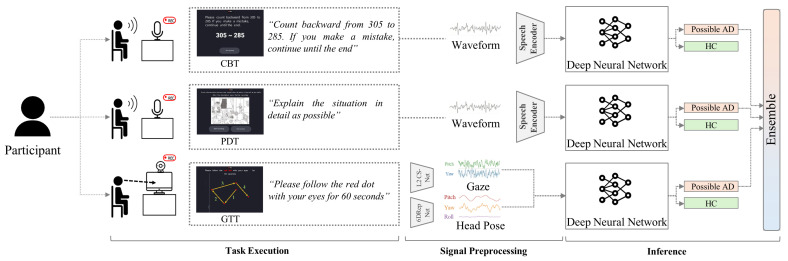
Overview of the proposed multi-modal sensing framework for AD detection. Participants perform three complementary cognitive tasks: Counting Backward (CBT), Picture Description (PDT), and Gaze Tracking (GTT). During task execution, speech and visual signals are recorded and preprocessed to extract modality-specific features, including pretrained acoustic representations and visual cues (gaze and head pose). Task-specific deep neural networks independently predict AD likelihood, and the final diagnosis is obtained by an ensemble algorithm that integrates these complementary predictions, enhancing robustness and sensitivity for real-world screening.

This paper proposes a multi-modal sensing framework that integrates complementary acoustic and visual cues for the detection of AD, as illustrated in Figure 2. In this framework, each participant performs three distinct cognitive tasks, during which speech and gaze-related behavioral signals are recorded. These multi-modal signals are processed through task-specific pipelines, where dedicated deep neural networks independently estimate the likelihood of AD. The resulting predictions are subsequently integrated via an ensemble algorithm to produce a unified diagnostic decision, enabling robust and sensitive detection across heterogeneous cognitive manifestations.

### 4.1. Deep Neural Networks

The recorded speech and visual signals are processed by dedicated deep neural networks to estimate task-specific AD likelihoods. Each modality is handled by a tailored model pipeline that captures its unique characteristics. All models are trained in a supervised learning setting using clinically labeled data, where each participant is assigned an AD or HC label based on clinical diagnosis. This design enables effective specialization for each sensor type while preserving a coherent and comparable decision structure across tasks.

#### 4.1.1. Speech-Based Model

For the speech-based tasks (CBT and PDT), we first extract acoustic features using pretrained models selected according to the nature of each task. Specifically, VGGish features are used for the CBT, while wav2vec features are used for the PDT. VGGish [61], trained on large-scale audio datasets, effectively captures general acoustic patterns and temporal characteristics in structured speech [62], making it suitable for CBT. In contrast, wav2vec [63] learns contextualized representations from raw audio through self-supervised learning, enabling richer modeling of spontaneous speech, which aligns with the nature of PDT.

We employ the same neural architecture for both tasks while training separate models. Since speech duration varies across participants, all input sequences are standardized to a fixed length via padding or truncation. CBT inputs consist of 128-dimensional VGGish features, whereas PDT inputs are 768-dimensional wav2vec features. The only architectural difference lies in the input projection layer, which adapts to the feature dimensionality, while the remaining architecture is identical. The features are projected into an embedding space and processed using a self-attention mechanism, followed by convolutional blocks and a bidirectional LSTM. Demographic information, specifically age and gender, was incorporated by concatenation with the learned representation, while education and MMSE were not included. Finally, each model performs classification to distinguish between HC and AD.

It is important to note that classical machine learning classifiers (e.g., Support Vector Machines) generally require fixed-length feature representations, which would necessitate additional temporal pooling or aggregation when applied to the sequential embeddings produced by the SSL encoder. Such preprocessing may reduce the temporal resolution of the original speech representations and potentially limit the model’s ability to capture fine-grained acoustic dynamics, including pauses, hesitations, and variations in speaking rate. Since our objective was to preserve and exploit temporal information contained in the sequential embeddings, we adopted a deep architecture with self-attention and BiLSTM modules that can directly model variable-length temporal sequences without requiring an explicit fixed-length transformation.

#### 4.1.2. Gaze-Based Model

For the GTT, we extract gaze and head pose features from facial video recordings, as patients with Alzheimer’s disease exhibit characteristic differences in head movement during gaze tracking tasks [64,65]. For each frame, the facial region is detected using RetinaFace [66], cropped, and resized to 224×224, forming a facial image sequence. This sequence is processed using two pretrained networks: L2CS-Net [67] for gaze estimation and 6DRepNet [68] for head pose estimation, resulting in 1D gaze and head pose sequences.

The detailed implementation of the GTT pipeline follows our previous work [65]. We employ a ResNet-based convolutional neural network to model gaze and head pose dynamics. The extracted sequences are converted into image-like representations and used as two input streams. The model consists of two parallel convolutional branches that process gaze and head pose features separately. Gaze features are fed into the main branch, while head pose features, which contain relatively less information, are processed through an auxiliary branch with reduced channel capacity. The resulting representations are then concatenated with demographic information, including age and gender but excluding education and MMSE levels. Finally, the model performs classification to distinguish between HC and AD.

### 4.2. Ensemble Methods

To integrate task-wise predictions across multiple cognitive domains, we evaluated three decision-level ensemble strategies: majority voting, soft voting, and any-positive aggregation. Each task-specific model independently outputs the probability of AD, and these predictions are subsequently combined to generate the final diagnosis.

For majority voting, each task prediction is first binarized using a threshold of 0.5. The final label is then determined by the majority class among the three task predictions. In soft voting, the predicted AD probabilities from all task-specific models are averaged with equal weights, and the final prediction is assigned using a threshold of 0.5 on the aggregated probability. No additional probability calibration was applied prior to aggregation.

The any-positive strategy classifies a participant as AD if at least one task-specific model predicts AD. This conservative decision rule is designed to prioritize sensitivity and minimize missed AD cases, reflecting the requirements of real-world screening scenarios where overlooking potential patients is clinically undesirable.

Unlike feature-level fusion approaches, the proposed framework performs decision-level aggregation across independently trained task-specific models. Since each task captures distinct cognitive domains, this strategy enables complementary behavioral evidence to be integrated while maintaining modularity and interpretability.

### 4.3. Evaluation Metrics

To evaluate the performance of the proposed model, five widely used classification metrics were employed: Accuracy, Sensitivity, Specificity, F1-score, and Area Under the Receiver Operating Characteristic Curve (AUC-ROC). These metrics provide complementary perspectives on the diagnostic capability of the model, particularly in medical classification tasks where class imbalance and false predictions are critical considerations.

Accuracy measures the overall proportion of correctly classified samples among all samples:

(1) $$\mathrm{Accuracy}=\frac{TP+TN}{TP+TN+FP+FN}$$

where TP, TN, FP, and FN denote true positives, true negatives, false positives, and false negatives, respectively.

Sensitivity, also referred to as recall or true positive rate, measures the ability of the model to correctly identify positive cases:

(2) $$\mathrm{Sensitivity}=\frac{TP}{TP+FN}$$

This metric is particularly important in medical diagnosis tasks, as it reflects the capability of detecting patients with the target condition.

Specificity measures the ability of the model to correctly identify negative cases:

(3) $$\mathrm{Specificity}=\frac{TN}{TN+FP}$$

A high specificity indicates that the model effectively reduces false positive predictions.

The F1-score is the harmonic mean of precision and recall, providing a balanced evaluation between false positives and false negatives:

(4) $$F1-\mathrm{score}=\frac{2\times \mathrm{Precision}\times \mathrm{Recall}}{\mathrm{Precision}+\mathrm{Recall}}$$

where precision is defined as:

(5) $$\mathrm{Precision}=\frac{TP}{TP+FP}$$

and recall corresponds to sensitivity.

In the context of cognitive impairment screening, sensitivity is particularly important because failing to detect affected individuals may lead to delayed clinical intervention. However, optimizing sensitivity alone may substantially increase false positive predictions, potentially reducing the practical usability of the screening system. Therefore, the F1-score was additionally considered as an important metric because it provides a balanced assessment of precision and recall, enabling evaluation of the trade-off between missed detections and excessive false alarms.

The Area Under the Receiver Operating Characteristic Curve (AUC-ROC) evaluates the overall discriminative ability of the model across different classification thresholds. The ROC curve is generated by plotting the true positive rate against the false positive rate:

(6) $$\mathrm{False}\;\mathrm{Positive}\;\mathrm{Rate}=\frac{FP}{FP+TN}$$

An AUC value closer to 1 indicates superior classification performance, while a value near 0.5 suggests random guessing.

### 4.4. Leave-One-Subject-Out (LOSO) Cross-Validation

To evaluate the generalization capability of the proposed model across different subjects, Leave-One-Subject-Out (LOSO) cross-validation was employed. In LOSO evaluation, data from one subject were used as the test set, while data from all remaining subjects were used for training. This process was repeated iteratively until every subject had been used once as the test subject.

LOSO is advantageous in small-scale datasets, as it helps reduce performance variability caused by dataset composition and enables a more reliable evaluation across subjects. Compared to random sample-level splitting, LOSO provides a more realistic assessment of model performance in practical clinical scenarios where the model encounters unseen individuals.

For each LOSO fold, the training subjects were further divided into a training set and a validation set. The training set was used for model optimization, while the validation set was used for selecting the best-performing model.

The final performance metrics were computed by pooling the predictions from all individual LOSO folds and calculating the evaluation metrics on the complete aggregated set of predictions.

## 5. Results and Discussion

This section presents a comprehensive analysis of AD detection performance, progressing from uni-modal to multi-modal sensing. First, we evaluate the individual diagnostic capability of speech and visual cues across the three cognitive tasks. Second, we investigate the reliability and robustness of clinical decisions obtained from multi-modal sensing integration. Finally, we discuss the implications of the proposed framework for clinical decision-making, with a particular focus on its ability to reduce missed diagnoses and support practical, real-world screening.

### 5.1. Experimental Setup

All experiments were conducted under the Leave-One-Subject-Out (LOSO) cross-validation framework to evaluate the generalization capability across unseen subjects. For each LOSO fold, the training subjects were further divided into a training set (80%) and a validation set (20%). The validation set was used for model selection, and the model achieving the highest validation accuracy was selected for evaluation on the held-out test subject.

The proposed framework was evaluated using Accuracy (Acc.), Sensitivity (Sens.), Specificity (Spec.), F1-score, and Area Under the Receiver Operating Characteristic Curve (AUC-ROC).

Different optimization strategies were employed depending on the task modality. Cross-entropy loss was adopted for all classification tasks, and all experiments were conducted on a single NVIDIA A100 80GB GPU. The models were implemented in Python (v3.9.23) using the PyTorch framework (v2.5.0). Since each task employed different model architectures and input modalities, task-specific training configurations and hyperparameters were independently optimized. Detailed implementation settings for each task are summarized in Table 2.

### 5.2. Single-Task Analysis

We first analyze the diagnostic capability of individual tasks and sensing modalities to understand their respective contributions to AD detection. Overall, the results indicate that CBT provides the strongest unimodal diagnostic performance, while GTT offers complementary behavioral evidence that is highly competitive with speech-based tasks.

Table 3 presents the performance of two speech-based tasks on the speech cohort: the cognitive overloading task (CBT) and the spontaneous speech task (PDT). Compared to PDT, CBT achieves higher specificity, leading to improvements in both overall accuracy and F1-score. This suggests that cognitively demanding tasks enhance discriminability by eliciting more pronounced deficits, while still maintaining sensitivity to AD-related impairments.

As shown in Table 4, CBT consistently demonstrates the strongest performance across all three tasks, achieving the highest sensitivity on the speech-gaze subset. This can be attributed to the cognitive domain probed by CBT, which primarily assesses executive function and sustained attention through structured mental operations. Such cognitively demanding processes are often affected early in AD, making task-induced impairments more explicitly observable. This suggests that CBT is particularly effective as a unimodal sensing task for detecting AD, particularly in reducing missed cases.

In contrast, PDT primarily assesses language ability and semantic processing through spontaneous speech production, requiring participants to retrieve semantically relevant words, organize scene information, and produce coherent verbal descriptions. Similarly, GTT captures oculomotor control and visual attention by requiring participants to visually track moving stimuli on the screen, allowing the framework to assess responsiveness, attentional shifts, and coordinated gaze-head movements. Although PDT exhibits relatively lower performance, GTT shows competitive results relative to CBT and outperforms PDT, highlighting the diagnostic value of visuospatial attention and eye-movement abnormalities. Since speech and visual signals reflect distinct cognitive domains, they provide complementary perspectives for capturing AD-related impairments.

In summary, these results demonstrate that gaze-based behavioral cues are as informative as speech-based signals from a complementary viewpoint. This observation supports the necessity of a multi-modal sensing framework, where integrating heterogeneous modalities can improve diagnostic reliability and robustness.

### 5.3. Multi-Task Analysis

We next evaluate the effectiveness of integrating multiple sensing modalities across tasks, focusing on how ensemble strategies may improve diagnostic performance and reduce missed AD cases. This analysis highlights the potential benefit of combining heterogeneous cognitive cues for more robust clinical decision-making.

**Figure 3:**
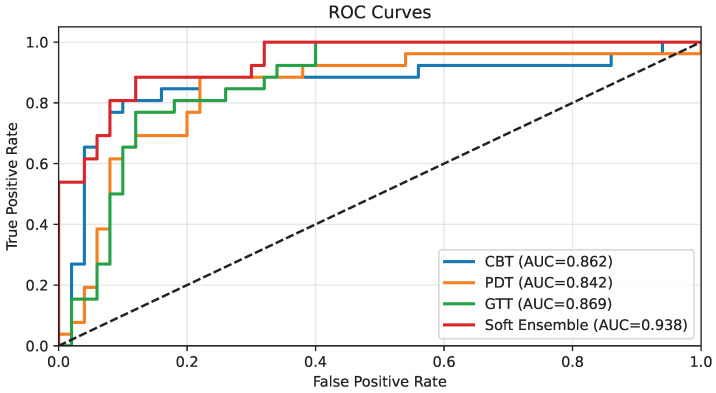
Receiver Operating Characteristic (ROC) curves comparing the three single-task models (CBT, PDT, and GTT; Table 4) with the soft-voting ensemble model (Table 5) on the speech-gaze subset. The dashed diagonal line represents the performance of a random classifier (AUC = 0.5).

Table 5 presents the performance of ensemble methods that integrate predictions from multiple sensing pipelines across the three tasks. Overall, multi-domain sensing integration shows a trend toward improved diagnostic performance compared with the best single-task model (CBT), although several performance metrics exhibit overlapping bootstrap confidence intervals. In particular, the soft-voting ensemble, which aggregates AD likelihoods across tasks, achieved the highest point estimates for most evaluation metrics and yielded an AUC of 0.938. These findings suggest that combining complementary information from multiple cognitive domains may provide additional diagnostic value while maintaining balanced performance. As illustrated in Figure 3, the ROC curves indicate improved discriminative ability for the soft-voting ensemble relative to the individual task-specific models.

Importantly, to address our primary objective of minimizing false negatives, we further evaluated an any-positive ensemble strategy, which classifies a participant as AD if any task predicts a positive outcome. This conservative decision rule increased sensitivity to 92.31%, resulting in only 7.69% (two subjects) of AD cases being missed across the entire evaluation. Although this gain in sensitivity was accompanied by reduced specificity, the approach maintained reasonable overall diagnostic performance. These findings suggest that multi-domain integration can support sensitivity-oriented screening by capturing complementary cognitive impairments across different tasks, thereby reducing the likelihood of missed AD cases.

### 5.4. Reliability as an AD Screening Tool

Conventional cognitive screening tools, such as the Mini-Mental State Examination (MMSE) and the Montreal Cognitive Assessment (MoCA), are widely adopted for AD detection due to their simplicity and clinical interpretability. Both tests demonstrate strong discriminative ability for AD, with reported AUC ranges of 0.87–0.99 for MoCA [69,70] and 0.67–0.99 for MMSE [70,71], and mean AUCs of approximately 0.84 and 0.82, respectively [72]. However, these assessments require structured administration and scoring by trained clinicians, introducing constraints in terms of time, cost, and scalability. In practice, their performance may also vary depending on the examiner’s expertise, patient cooperation, and contextual factors.

In contrast, the proposed framework eliminates the need for clinician-dependent administration by leveraging automatically collected behavioral signals. Through speech and gaze sensing, the system enables fully automated screening using readily available devices, making it more suitable for large-scale and real-world deployment. Notably, the any-positive ensemble achieves a sensitivity of 92.31%, which is competitive with conventional screening tools. This high sensitivity is particularly important for screening applications, where missing AD cases must be minimized. Importantly, this sensitivity gain is achieved while maintaining 68.00% specificity, ensuring that improved case detection does not lead to excessive false positives.

Furthermore, the soft-voting ensemble provides a balanced alternative, achieving 88.46% sensitivity with strong overall performance. By integrating complementary behavioral cues across multiple cognitive domains, the proposed framework captures heterogeneous manifestations of cognitive impairment that may not be consistently observable through a single, clinician-administered test.

Overall, the proposed system offers a practical and scalable alternative to conventional screening tools. By removing the dependency on expert administration while achieving high sensitivity, it aims to enable accessible, continuous, and non-invasive AD screening in future real-world environments, thereby facilitating earlier screening opportunities and timely clinical intervention.

### 5.5. Clinical Trade-Offs for AD Screening

The primary objective of AD screening is not merely to achieve high overall accuracy, but to reliably identify all individuals at risk, particularly at early stages where intervention can meaningfully delay disease progression. In this context, minimizing false negatives is of paramount importance, as missed AD cases may continue to deteriorate without timely clinical attention, leading to irreversible cognitive decline. Motivated by this principle, the proposed multi-domain behavioral sensing framework is designed to capture heterogeneous cognitive impairments by probing multiple domains through complementary tasks and modalities. The soft-voting strategy demonstrates strong overall screening performance by aggregating probabilistic evidence across tasks, reflecting a balanced integration of diverse cognitive cues.

However, practical screening often prioritizes sensitivity over balanced accuracy, especially in broad screening scenarios where missing cases must be minimized. From this perspective, the any-positive ensemble strategy serves as a conservative yet clinically meaningful decision rule, ensuring that potential AD cases are flagged whenever any cognitive domain exhibits abnormality. This approach aligns with established clinical screening practices, where initial assessments favor inclusivity to avoid overlooking at-risk individuals. Notably, our framework achieves substantially higher sensitivity compared to traditional screening tools such as MMSE and MoCA, while still maintaining a reasonable specificity of 68.00%. This level of specificity is sufficient to limit excessive false positives, thereby reducing unnecessary follow-up diagnostic procedures and associated clinical burden.

Importantly, the proposed framework introduces a flexible trade-off between sensitivity and specificity through its ensemble design, allowing adaptation to different clinical scenarios. For instance, sensitivity-oriented configurations (e.g., any-positive) are suitable for large-scale population screening, whereas more balanced strategies (e.g., soft voting) may be preferred in secondary assessments. By explicitly modeling heterogeneous cognitive manifestations across multiple domains, the framework mitigates the risk of compensatory behaviors that may mask impairments in single-task settings. As a result, it provides a more comprehensive and reliable assessment of cognitive status.

Overall, these findings suggest that multi-domain sensing approaches are not only beneficial but necessary for practical AD screening. By prioritizing sensitivity while maintaining acceptable specificity, the proposed framework offers a clinically viable solution for early detection, enabling timely intervention and supporting scalable deployment in future real-world healthcare environments.

### 5.6. Ablation Study on Demographic Information

Since demographic imbalance existed between the healthy control group and the patient group, particularly in terms of age and sex distribution, an additional ablation study was conducted to investigate whether the proposed model primarily relied on demographic information rather than biomarker-derived features for classification.

In the main experiment, demographic metadata consisting of age and sex were included together with the biomarker features. To assess the contribution of behavioral biomarkers beyond these demographic variables, we additionally evaluated the model after excluding all demographic information from the input features. Furthermore, a demographic-only baseline using age and sex alone was evaluated under the same experimental protocol.

Specifically, the biomarker-only ablation experiment was performed using identical model architectures, training procedures, and LOSO cross-validation settings while removing age and sex information from the input features. The demographic-only baseline was evaluated using the same LOSO framework to quantify the predictive value of age and sex alone. The detailed results are presented in Table 6.

The results show that age and sex alone (Dem. Info. Only) provide meaningful discriminatory power, achieving an AUC of 0.794 and a sensitivity of 83.33%. This finding suggests that demographic characteristics contribute to disease classification in our cohort. However, incorporating behavioral biomarkers substantially improves overall diagnostic performance. Compared with the demographic-only baseline, the proposed multimodal framework consistently achieved higher accuracy, specificity, F1-score, and AUC across ensemble strategies.

When age and sex information were removed (W/o Dem. Info), the Majority Voting and Soft Voting approaches exhibited only moderate performance degradation, with Soft Voting maintaining an accuracy of 88.16% and an AUC of 0.890. These findings indicate that the proposed framework captures clinically relevant behavioral patterns that extend beyond demographic information alone. In contrast, the Any-Positive strategy showed a substantial reduction in specificity and overall accuracy after demographic information was excluded, suggesting that this sensitivity-oriented decision rule is more sensitive to variations in individual task-level predictions.

Overall, the ablation study demonstrates that age and sex contribute useful diagnostic information, but do not fully account for the performance of the proposed framework. The strong performance maintained after removing demographic information, together with the consistent improvement over the demographic-only baseline, supports the validity of the proposed speech- and gaze-derived biomarkers as informative indicators for Alzheimer’s disease screening.

### 5.7. Limitations and Future Works

The proposed framework demonstrates the potential of non-invasive, easily accessible behavioral signals for scalable and cost-effective screening. However, several limitations remain. The relatively small sample size may limit the generalizability of the findings. In addition, since not all participants agreed to facial video recording, the multi-modal analysis was conducted on a reduced subset of participants, which may introduce potential selection bias. A post hoc comparison indicated that approximately half of the participants declined video recording across groups, while relatively higher consent rates were observed among cognitively normal male participants. No substantial differences were observed in other demographic characteristics. Nevertheless, such consent-related sampling bias may still influence the representativeness and generalizability of the multi-modal findings.

The current framework also processes each task independently and does not explicitly model cross-task interactions, which could further enhance performance. Despite the availability of various non-invasive biomarkers, this study focused primarily on speech and gaze modalities due to their accessibility. Future work should explore additional behavioral and physiological signals to further enhance diagnostic performance. Furthermore, collecting larger and more diverse datasets across multiple environments will be essential for improving the robustness and generalizability of the proposed framework.

## 6. Conclusions

In this paper, we proposed a multi-domain behavioral sensing framework for AD diagnosis based on non-invasive behavioral biomarkers. Existing approaches often rely on a single modality, which limits their ability to capture the heterogeneous nature of cognitive impairment. To address this limitation, we designed a framework that integrates multiple cognitive tasks, including speech-based and gaze-based assessments.

The proposed approach leverages task-specific models and ensemble strategies to effectively combine complementary information from different modalities. By incorporating both speech and gaze signals, the framework enables a more comprehensive analysis of cognitive function while maintaining scalability through the use of non-invasive and easily accessible data sources.

Experimental results demonstrated that each individual task provides reliable diagnostic performance, and ensemble methods further improve overall performance and robustness. In particular, the any-positive ensemble achieved high sensitivity, highlighting its potential for minimizing missed cases in clinical screening scenarios. These findings indicate the effectiveness of integrating multiple behavioral signals for Alzheimer’s disease detection.

Despite these promising results, several limitations remain. The current framework performs multi-task diagnosis through task-level independent models followed by ensemble aggregation, rather than using a unified architecture that jointly models feature interactions across tasks. In addition, the present study focuses on only two types of behavioral biomarkers, speech, and gaze. Future work will aim to incorporate additional sensing modalities and develop an integrated model that jointly reasons over cross-task features to further enhance the performance, robustness, and clinical reliability of the proposed framework.

## Figures and Tables

**Table 1:** Demographic characteristics of the study participants. The speech cohort includes all participants who completed the speech-based tasks (Counting Backward Task and Picture Description Task), whereas the speech-gaze subset represents participants who additionally completed the gaze tracking task. All variables except gender are expressed as mean ± standard deviation.

Variable	Speech Cohort (*n* = 128)		Speech-Gaze Subset (*n* = 76)	
	HC (*n* = 77)	AD (*n* = 51)	HC (*n* = 50)	AD (*n* = 26)
Age	66.0 ± 8.1	77.6 ± 6.9	66.3 ± 8.8	79.4 ± 6.6
Education ^1^	13.3 ± 3.7	10.1 ± 5.4	13.5 ± 3.2	9.3 ± 5.8
Gender ^2^	29/48	8/43	24/26	4/22
MMSE	28.9 ± 1.3	24.0 ± 4.7	28.8 ± 1.3	23.1 ± 5.3

^1^ Years of education; ^2^ male/female.

**Table 2:** Training configurations for each single-task model, including optimizer, learning rate, batch size, number of epochs, and weight decay.

Task	Optimizer	Learning Rate	Batch Size	Epochs	Weight Decay
CBT	AdamW	$2\times 10^{-3}$	64	250	0.01
PDT	AdamW	$2\times 10^{-4}$	32	150	0.01
GTT	SGD	$1\times 10^{-4}$	8	100	-

**Table 3:** Performance comparison across speech tasks on the 128 participants who completed the speech tasks. Values in parentheses indicate the 95% bootstrap confidence intervals.

Task	Acc. (%)	Sens. (%)	Spec. (%)	F1-Score (%)	AUC
CBT	**83.59** (77.34–89.06)	**78.43** (67.35–89.66)	**87.01** (78.95–93.75)	**79.21** (69.56–87.13)	**0.883** (0.817–0.941)
PDT	80.47 (73.44–86.72)	**78.43** (65.95–89.36)	81.82 (72.62–90.00)	76.19 (65.26–84.69)	0.846 (0.771–0.911)

Acc.: Accuracy; Sens.: Sensitivity; Spec.: Specificity. The best performance is highlighted in bold.

**Table 4:** Performance comparison across single-task models on the 76 participants who completed both speech and gaze tasks. Values in parentheses indicate the 95% bootstrap confidence intervals.

Task	Acc. (%)	Sens. (%)	Spec. (%)	F1-Score (%)	AUC
CBT	**84.21** (76.32–92.11)	**80.77** (65.22–95.24)	**86.00** (75.00–94.23)	**77.78** (63.83–88.58)	0.862 (0.743–0.955)
PDT	78.95 (69.74–86.87)	69.23 (51.57–86.96)	84.00 (72.91–93.19)	69.23 (53.33–81.97)	0.842 (0.732–0.929)
GTT	80.26 (71.05–88.19)	76.92 (58.33–91.67)	82.00 (71.11–91.89)	72.73 (56.60–84.51)	**0.869** (0.778–0.940)

Acc.: Accuracy; Sens.: Sensitivity; Spec.: Specificity. The best and second-best performances are highlighted in bold and underlined, respectively.

**Table 5:** Performance comparison across ensemble methods, evaluated on the 76 participants who completed both the speech and gaze tasks. Values in parentheses indicate the 95% bootstrap confidence intervals.

Ensemble Method	Acc. (%)	Sens. (%)	Spec. (%)	F1-Score (%)	AUC
Majority voting	85.53 (76.32–93.42)	84.62 (70.37–96.43)	86.00 (74.99–95.46)	80.00 (65.31–90.63)	- (-)
Soft voting	**88.16** (80.26–94.74)	88.46 (76.00–100.00)	**88.00** (78.25–96.08)	**83.64** (71.11–93.16)	**0.938** (0.878–0.981)
Any-positive	76.32 (67.11–85.53)	**92.31** (80.77–100.00)	68.00 (53.66–80.43)	72.73 (59.65–83.33)	- (-)

Acc.: Accuracy; Sens.: Sensitivity; Spec.: Specificity. The best and second-best performances are highlighted in bold and underlined, respectively.

**Table 6:** Performance comparison of ensemble strategies with and without demographic information on the 76 participants who completed both speech and gaze tasks. Values in parentheses indicate the 95% bootstrap confidence intervals.

Ensemble Method	Acc. (%)	Sens. (%)	Spec. (%)	F1-Score (%)	AUC
With Dem. Info.					
Majority voting	85.53 (76.32–93.42)	84.62 (70.37–96.43)	86.00 (74.99–95.46)	80.00 (65.31–90.63)	- (-)
Soft voting	**88.16** (80.26–94.74)	88.46 (76.00–100.00)	**88.00** (78.25–96.08)	**83.64** (71.11–93.16)	**0.938** (0.878–0.981)
Any-positive	76.32 (67.11–85.53)	**92.31** (80.77–100.00)	68.00 (53.66–80.43)	72.73 (59.65–83.33)	- (-)
W/o Dem. Info.					
Majority voting	82.89 (73.68–90.79)	80.77 (63.64–95.65)	84.00 (72.54–93.44)	76.36 (62.06–87.27)	- (-)
Soft voting	**88.16** (81.55–94.74)	84.62 (69.57–96.67)	**90.00** (80.77–97.73)	83.02 (70.26–92.54)	0.890 (0.783–0.967)
Any-positive	50.00 (38.16–60.53)	**96.15** (86.36–100.00)	26.00 (14.81–38.10)	56.82 (43.04–68.18)	- (-)
Dem. Info. Only					
Age & Sex	67.19 (58.59–75.78)	83.33 (60.00–100.00)	65.52 (56.52–74.34)	32.26 (15.79–46.58)	0.794 (0.681–0.895)

Acc.: Accuracy; Sens.: Sensitivity; Spec.: Specificity. The best and second-best performances are highlighted in bold and underlined, respectively.

## Data Availability

The data presented in this study are available on request from the corresponding author. The data are not publicly available due to data privacy.

## Abbreviations

The following abbreviations are used in this manuscript:

AD	Alzheimer’s disease
HC	Healthy control
CBT	Counting Backward Task
PDT	Picture Description Task
GTT	Gaze Tracking Task
